# Exploring the predictors of foreign language anxiety: the roles of language proficiency, language exposure, and cognitive control

**DOI:** 10.3389/fpsyt.2024.1492701

**Published:** 2024-11-28

**Authors:** Yan Xu, Zhilong Xie

**Affiliations:** ^1^ Foreign Languages School, Jinggangshan University, Ji’an, Jiangxi, China; ^2^ Foreign Languages College, Jiangxi Normal University, Nanchang, Jiangxi, China

**Keywords:** L2 proficiency, L2 exposure, cognitive control, FLA, English learners

## Abstract

This research delves into unexplored territories of Foreign Language Anxiety (FLA), going beyond the traditional focus on language proficiency. We examined the nuanced roles of language exposure and individual differences in cognitive control abilities in shaping FLA. By engaging 46 English learners in a comprehensive assessment, our analysis uncovered significant yet distinct contributions of these factors to various aspects of FLA. Notably, proficiency predicted communication and overall anxiety, exposure influenced evaluation anxiety, while inhibition and mental set shifting abilities significantly predicted communication and test anxiety respectively. These findings illuminate the complexity of FLA, revealing that it stems from a multifaceted interplay of language proficiency, exposure, and cognitive control. This holistic understanding offers valuable insights for educators and learners alike, emphasizing the importance of tailored strategies that address not just language proficiency but also exposure opportunities and cognitive strengths.

## Introduction

Foreign language anxiety (FLA) has garnered significant attention as an affective factor in second/foreign language acquisition. Prior research has consistently linked FLA to language proficiency, highlighting a negative correlation (1–6). While much emphasis has been placed on external sources of FLA, such as teacher-student dynamics and classroom environments (7), the investigation into the roles of language learning experiences and individual cognitive differences has been relatively scarce. Despite isolated studies exploring the relationships between language use experiences (8) and cognitive control abilities (9, 10) with anxiety in broader contexts, the intricate interplay between these factors and FLA remains underexplored. The present study endeavors to bridge this gap by delving into the specific contributions of language proficiency, language exposure, and cognitive control abilities to FLA, offering a nuanced perspective on the multifaceted nature of this affective variable.

### Anxiety and FLA

Spielberger et al. (11) articulated anxiety as a subjective experience encompassing tension, apprehension, nervousness, and worry, triggered by autonomic nervous system arousal. This emotion assumes two distinct forms: trait anxiety, a stable personality characteristic pervading various situations, and state anxiety, a fleeting emotional state contingent upon specific contexts (12). MacIntyre and Gardner (13) introduced the concept of foreign language classroom anxiety (FLCA), a particular manifestation rooted in the unique stressors of language learning environments, distinguishing it from generalized anxiety scenarios (3, 14, 15).

FLCA, as defined by Horwitz et al. (15), embodies a distinct blend of self-perceptions, beliefs, emotions, and behaviors that stem from the singular challenges of foreign language classrooms. This anxiety is multifaceted, transcending the mere sum of its components: communication apprehension, test anxiety, and evaluation anxiety. Communication apprehension encompasses the fear of engaging in linguistic interactions, whether it be speaking or listening. Test anxiety reflects the performance-related stress of failing linguistic assessments. Evaluation anxiety, meanwhile, arises from the anticipation of negative judgments from proficient teachers or peers, leading to an aversion towards evaluative situations. Recognizing the intricacies of FLCA, Horwitz et al. (15) devised the Foreign Language Classroom Anxiety Scale (FLCAS), a robust instrument with an alpha coefficient of 0.93, indicative of high internal reliability. Widely adopted and validated (16), the FLCAS comprises 33 questions, meticulously crafted to capture the nuanced dimensions of anxiety within foreign language classrooms. Given that language learning often unfolds primarily within classroom settings, the current study operationally defines foreign language anxiety (FLA) as synonymous with FLCA, focusing on the general experiences of learners within these environments.

### L2 proficiency and FLA

Language anxiety has emerged as a pivotal affective factor, consistently ranking among the foremost predictors of students’ linguistic proficiency (17). A vast majority of research endeavors have zeroed in on the intricate interplay between foreign language achievement and anxiety, yielding remarkably consistent results that underscore a moderately inverse relationship between the two (18). Azher et al. (19), for instance, surveyed 149 undergraduates using the FLCA questionnaire and correlated their language achievement, as indicated by GPA, with anxiety levels. Their analysis underscored a statistically significant negative correlation (*r*=-.273, *p*<.001). Similarly, a comprehensive meta-analysis by Teimouri et al. (6), spanning 105 samples across 23 countries and encompassing 19,933 participants, reaffirmed this negative link, with a stronger correlation coefficient of *r*=-.36 (*p*<.001).

Notably, the impact of language achievement extends across the entire foreign language learning continuum, from input to processing to output, all of which are inversely related to FLA, as evidenced by Onwuegbuzie et al. (20). While a substantial body of research has examined the overarching relationship between language proficiency and FLA, there is also a growing interest in exploring its nuanced connections with specific components of FLA. Su (21), for example, isolated the Communication Apprehension Subscale from the FLCAS to assess its relationship with English proficiency. Their findings mirrored a negative correlation (*r*=-.119, *p*<.001), indicating that higher self-perceived English proficiency is associated with lower communication anxiety. Likewise, Hu et al. (22), in a study involving 631 primary school students, uncovered a significant negative relationship between language achievement and all individual components of FLA (*p*s<.01), further corroborating the multifaceted nature of this connection. In conclusion, language proficiency stands as a pivotal factor influencing FLA, with its relationship to various FLA components warranting further investigation to deepen our understanding of this complex interplay.

### Language exposure and FLA

Language exposure is paramount to igniting and fostering linguistic prowess in early stages, as emphasized by Mayberry et al. (23). Acting as a pivotal predictor of vocabulary richness and grammatical mastery, the quantum of L2 exposure intimately correlates with the trajectory of bilingual development, fostering proficiency through dual language immersion (24). Conversely, inadequate L2 exposure hinders language acquisition progress, fostering anxiety and unease in learners during language production, thereby potentially exacerbating foreign language anxiety (FLA) (25). The influence of language exposure on FLA manifests in diverse contexts, including overseas experiences, immersive classrooms, and the age of acquisition (AoA). Overseas sojourns not only accelerate linguistic proficiency but also bolster mental resilience, with prior encounters with target cultures inversely predicting FLA (20, 26b). This underscores that EFL learners immersed in the target language environment exhibit diminished FLA. Similarly, immersion students, who acknowledge and manage their FLA more effectively than their non-immersion peers (27), report lower communication anxiety, with extracurricular language usage further mitigating FLA (28).

AoA also plays a crucial role, as early L2 exposure tends to diminish FLA (28, 29). Research on Chinese EFL learners reveals that earlier L2 exposure is inversely associated with FLA across linguistic contexts, with pre-school exposure leading to significantly lower anxiety levels (29). Similarly, American-born bilinguals, who acquired English at a young age, exhibit lower FLA compared to their counterparts residing outside the U.S.-Mexico border (30). In French immersion programs, heightened language interaction with advancing grades (7-9) correlates with declining FLA levels (31).

While some scholars posit an indirect link, where increased L2 proficiency stemming from exposure subsequently mitigates FLA (32), direct evidence elucidating the explicit impact of L2 exposure on FLA remains elusive. Further research is warranted to unravel the intricate interplay between these variables and its implications for language learning outcomes.

### Cognitive control and FLA

Cognitive control, an umbrella term encompassing processes like inhibition, mental set shifting, and working memory updating (33, 34), is instrumental in goal-directed behaviors. Language learning, inherently a cognitive endeavor involving encoding, storage, and retrieval (35), has recently sparked interest in its interplay with cognitive control and anxiety. Research underscores a bidirectional relationship between cognitive control and anxiety. High anxiety levels have been associated with cognitive impairments, including deficits in cognitive control (36), while impairments in cognitive control, particularly inflexibility, can precipitate anxiety (10, 37–39). Notably, various facets of cognitive control negatively correlate with anxiety (40–42), highlighting the potential for a vicious cycle where cognitive difficulties fuel anxiety, and vice versa.

In the context of language learning, particularly foreign language acquisition, cognitive control takes on heightened significance. When two languages are simultaneously activated, the ability to inhibit the non-target language, especially the dominant one, becomes crucial for effective communication (43). Furthermore, cognitive control facilitates context monitoring and seamless language switching (44), suggesting that enhanced cognitive control abilities may foster a more relaxed language learning experience, thereby mitigating foreign language anxiety (FLA). While prior studies have delved into the relationship between cognitive control and anxiety in general settings, their specific role in the foreign language learning context remains underexplored. We posit that robust cognitive control abilities expedite foreign language processing, fostering emotional adaptability and ultimately mitigating FLA.

### The current study

Based on the above review, the current study seeks to bridge a significant gap in understanding the intricate relationship between second language (L2) proficiency, exposure to the L2 environment, and cognitive control abilities, and their differential contributions to foreign language anxiety (FLA) among learners of English as a Foreign Language (EFL). The study aims to investigate three distinct research questions (RQs).

RQ1: To what extent does second language (L2) proficiency serve as a predictor of foreign language anxiety (FLA)?

RQ2: Is second language (L2) exposure a potential indicator of foreign language anxiety (FLA)?

RQ3: Does cognitive control act as a predictor of foreign language anxiety (FLA)?

Our central hypothesis posits that while L2 proficiency, exposure, and cognitive control abilities are all vital factors that contribute to FLA, they each exert their influence in distinct and nuanced ways. L2 proficiency, often seen as a cornerstone of language learning, may mitigate FLA by enhancing learners’ confidence and reducing their fear of making mistakes. L2 exposure, which refers to the amount and quality of interaction with the target language, can either exacerbate or alleviate FLA depending on the learner’s perception of their language environment and their level of preparedness to engage in such interactions. Cognitive control abilities, encompassing executive functions such as inhibition and mental set shifting, play a pivotal role in modulating FLA. They enable learners to effectively manage distractions, prioritize tasks, and switch their mental sets during language learning activities. By strengthening these abilities, learners may be better equipped to cope with anxiety-provoking situations and persist in their language learning endeavors. Therefore, the current study aims to make a significant contribution to the field of language learning and anxiety research by providing a nuanced understanding of the differential contributions of L2 proficiency, exposure, and cognitive control abilities to FLA. By integrating these multifaceted factors and offering practical insights, our research endeavors to pave the way for more effective strategies to alleviate FLA and foster successful language learning experiences for EFL learners worldwide.

## Materials and methods

### Procedure

All participants were required to complete a series of tests in a single session within a quiet laboratory setting. Initially, they undertook two cognitive control tasks: the Flanker Task and the Wisconsin Card Sorting Test. Subsequently, they completed the Foreign Language Classroom Anxiety Questionnaire. Following this, they reported their language background, specifically their experiences and proficiency in learning English as a foreign language, using the Language Experience and Proficiency Questionnaire. Finally, they took an objective L2 (English) proficiency test, the Semantic Category Verbal Fluency Test. The entire testing process took approximately one hour, with the sequence of tests being counter-balanced to control for order effects.

### Participants

Forty-six (44 females, 2 males) English major postgraduate students from Jiangxi Normal University participated in the current study. All of them voluntarily participated in the study for course credit with informed consent, and their rights were protected by the protocols of the Academic Committee of the university. The participants were native speakers of Chinese and took English as a foreign language, which is mandatory in most schools in China. They started English learning at about 10 years old in schools. They were English major students in their first year of the master’s program. In their previous college years, they studied a series of English courses, such as Listening, Speaking, Reading, Writing, British Literature, American Literature, Linguistics, Language Teaching, and Translation Studies.

## Materials

### Linguistic background

*The Language Experience and Proficiency Questionnaire (LEAP-Q)* was used to gather participants’ linguistic backgrounds, including age, language learning history, education, language proficiency and language exposure (45, 46). LEAP-Q encompasses four fundamental language skills: listening, speaking, reading, and writing, assessed separately for both L1 and L2. Each skill is rated on a scale from 1 to 10, where “1” signifies minimal proficiency in the language and “10” represents native-level competence. Language exposure is calculated as a percentage, based on the fact that participants utilized either their first language (L1) or their second language (L2), with the sum of L1 and L2 equaling 100%.

### Objective L2 proficiency

In order to objectively assess L2 proficiency, we employed the Semantic Category Verbal Fluency Test, a widely recognized measure of vocabulary size (44) and a commonly utilized proxy for overall proficiency in L2 acquisition (47–49). In comparison to subjective self-assessments, objective tests are considered more reliable, despite their high correlation (46). During the test, participants were instructed to generate as many words as possible within 60 seconds for three specific categories: job titles, furniture items, and fruits. The total number of words reported by each participant was tallied and used as their test score. It is worth noting that we did not assess participants’ verbal fluency in Chinese (L1), as it is their native and primary language. Furthermore, all participants had received higher education, which typically requires a high level of proficiency in Chinese. Consequently, their first language (L1) proficiency was deemed uniformly high, despite potential individual variations.

### Foreign language anxiety

We adopted the *Foreign Language Classroom Anxiety Scale* (15) as the operational measure of foreign language anxiety (FLA) since our participants were students in college and they used English primarily in English classes. As previously stated, the scale has gained widespread recognition within the field for its reliable assessment of various dimensions of foreign language anxiety. This assessment is comprised of 33 questions, each utilizing a 5-point Likert scale that ranges from 1, indicating “strongly disagree,” to 5, representing “strongly agree.” Of the 33 questions, the items that reflect lack of anxiety include 2, 5, 8, 11, 14, 18, 22, 28, and 32, whereas all the other 24 items reflect anxiety. Consequently, the nine items that indicate a deficiency in anxiety should have their scoring inverted, ensuring that elevated scores correspond to heightened levels of foreign language anxiety. Overall, the questions were categorized into four aspects: communication apprehension, test anxiety, negative evaluation, and general learning. Communication apprehension includes 12 items: 1, 3, 4, 9, 13, 14, 18, 20, 24, 27, 29, and 33. Test anxiety includes 3 items: 8, 10, and 21. Fear of negative evaluation includes 6 items: 2, 7, 15, 19, 23, and 31. The remaining items are labeled as general anxiety.

### Cognitive control

*The Flanker Task* (50) is a typical and well-established behavioral task used to measure cognitive control, particularly for inhibition. Inhibition reflects the ability to suppress responses that are inappropriate in a given situation (33). In the task, participants are required to respond to the direction of the target stimuli by pressing designated keys on the computer. The target stimulus is a red arrow flanked by three types of shapes. In the first type (congruent condition), the target arrow is flanked by arrows of the same direction; in the second type (neutral condition), the target arrow is flanked by diamond symbols; in the third type (incongruent condition), the target arrow is flanked by arrows of opposite direction. In the E-prime designed computerized procedure following previous literature (51–53), the task was designed in two blocks: a practice block and a formal experimental block. For each trial, a fixation of “+” appeared for 250 ms, and then a randomly presented stimulus appeared for 2000 ms, within which participants were to decide the direction of each target arrow as fast and accurately as possible by pressing designated keys on the computer keyboard. The participants could not start the formal experimental block (108 trials) unless they performed with an accuracy rate above 80% in the practice block.

The *Wisconsin Card Sorting Test (WCST)* is a well-recognized behavioral task used to measure mental set shifting (53–55). Mental set shifting is the ability to switch back and forth between multiple tasks, operations, dimensions, or mental sets (33, 56). In this test, participants are asked to classify each response card according to four stimulus cards (one red triangle, two green stars, three yellow crosses, and four blue circles). Each card is a mixture of numbers, colors, and shapes. In the computerized version of WCST via E-prime 2.0, there were 12 practice trials and 128 formal experimental trials. In each trial, a fixation “+” appeared for 1000 ms. Then the four stimulus cards and a response card appeared simultaneously. Each participant was required to classify the response card according to any of the stimulus cards by pressing designated keys (within 3000 ms), followed by feedback for 1000 ms before the next trial. The implied sorting rule would change after a few trials (5-9 trials) without the participants’ awareness.

## Results

All the data and data analysis syntax are accessible at https://osf.io/vpzrd/(DOI 10.17605/OSF.IO/VPZRD). In our study’s data analyses, we begin by presenting the descriptive statistics for all participants’ background information. Subsequently, we detail the descriptive data regarding participants’ foreign language anxiety levels and their cognitive control performance metrics. Lastly, we unveil the outcomes of our correlation and regression analyses, aimed at identifying which variables are predictive of the various facets of foreign language anxiety.

### Linguistic backgrounds

The descriptive data presented in **Table 1** indicated that the average age of the participants (*N*=46) was 23.57 years (*SD*=1.36). They had acquired English as a foreign language for an average duration of 13.50 years (*SD*=1.82). For the entire group, the exposure to English amounted to 32.17% (*SD*=13.48) of their linguistic experience. Consequently, exposure to Chinese constituted 67.83% for our participants. The mean English verbal fluency across the three categories (job, furniture, fruits) was 31.09 (*SD*=5.99), serving as an objective measure of vocabulary size and, by extension, language proficiency. Self-reported language proficiency ratings revealed an average score of 21.41 (SD=2.17) for English and 35.17 (SD=2.63) for Chinese. While the participants demonstrated proficiency in both languages, these data suggest that Chinese is their dominant language.

**Table 1:** Descriptive data of participants’ linguistic information .

	N	Min	Max	M	SD
Age	46	21.00	28.00	23.57	1.36
L2 Learning History (yrs)	46	10.00	17.00	13.50	1.82
Education (yrs)	46	15.00	22.00	17.57	1.13
English Exposure (%)	46	5.00	70.00	32.17	13.48
English Verbal Fluency	46	19.00	45.00	31.09	5.99
English Proficiency (0-40)*	46	17.00	26.00	21.41	2.17
Chinese Proficiency (0-40)	46	28.00	40.00	35.17	2.63

The total score of self-rated language proficiency is 40, with each skill (listening, reading, speaking, and writing) for 10 points.

### Foreign language anxiety

As mentioned in the method section, the *Foreign Language Classroom Anxiety Scale* (15) assesses general anxiety, communication apprehension, test anxiety, and evaluation anxiety, with further item breakdowns shown in **Table 2**. As evident from **Table 2**, the participants collectively exhibited an overall anxiety level of 88.04 (SD=8.54) out of a possible 165 points, suggesting a moderately high level of foreign language anxiety. More specifically, the level of communication apprehension is 32.43 out of 60 (54%), fear of negative evaluation 17.02 out of 30 (57%), test anxiety 9.24 out of 15 (62%), and general anxiety 29.35 out of 60 (49%). Upon examining the comprehensive data, it shows that, comparatively, test anxiety exhibits the highest degree of anxiety across the four categories, succeeded by fear of negative evaluation, communication apprehension, and general anxiety.

**Table 2:** Descriptive data of foreign language anxiety among participants.

	N	Min	Max	M	SD
General Anxiety	46	22.00	36.00	29.35	2.87
Communication Apprehension	46	20.00	42.00	32.43	4.91
Fear of Negative Evaluation	46	11.00	25.00	17.02	2.75
Test Anxiety	46	5.00	12.00	9.24	1.64
Overall Anxiety (total scores)	46	70.00	107.00	88.04	8.54

### Cognitive control tasks

In the Flanker task, two participants were excluded as a result of operation failure. In data trimming, for the response times (from the Flanker task and the WCST), outliers and deviations from 3SDs for each participant on each condition were excluded, accounting for about 2.1% of all data sets. The Flanker task was employed to assess inhibition, typically quantified by the difference in response times (RTs) between incongruent and congruent trials (57–59). In the present study, however, inhibition is computed using the formula “(incongruent - congruent)/congruent,” which represents the relative disparity in RTs between incongruent and congruent trials against the backdrop of baseline congruent RTs. This approach is beneficial in accounting for individual variances in overall processing speed. More modest outcomes indicate superior inhibition. The Wisconsin Card Sorting Test (WCST) was employed to assess mental set shifting (55). This is gauged by the quantity of categories successfully completed, with one category indicating that the participant has correctly completed at least five consecutive trials. A higher number of completed categories suggests a superior capacity for mental set shifting. The descriptive results of the two tasks are presented in **Table 3**.

**Table 3:** Descriptive data of cognitive control among participants.

	N	Min	Max	M	SD
Flanker Task (RTs)					
Congruent	44	382	784	510	75
Neutral	44	411	836	525	76
Incongruent	44	420	856	562	85
Inhibition	44	.02	.37	.10	.06
WCST					
Completed Categories	46	2.00	13.00	6.90	2.57

As **Table 3** shows, participants responded more quickly in the congruent condition than the neutral condition (*M*= 510 ms, *SD*=75 vs. *M*= 525 ms, *SD*=76), and the neutral condition was faster than the incongruent condition (*M*= 525 ms, SD=76 vs. *M*= 562 ms, *SD*=85). The indicator of inhibition was *M*=.10 (*SD*=.06). Repeated measures of variance analyses revealed that the factor of condition (congruent, neutral, and incongruent) was significant, *F* (2, 86) = 65.231, *p* <.001, *η* ^2^ =.603, reflecting that participant responded fastest in the congruent condition where there is a facilitation of the Flankers (<<**<**<<), whereas slowest in the incongruent condition where the Flankers conflict the direction of the target arrow (<<**>**<<). In the WCST, participants correctly completed 6.90 categories (*SD*=2.57). All these completed categories reveal how participants were able to switch mental sets when different rules were implied.

### Correlation analyses

In order to find out the relationship between participants’ linguistic background, foreign language proficiency, cognitive control, and foreign language anxiety, we conducted correlation analyses. The results of correlations between linguistic background, language proficiency, and foreign language anxiety are presented in **Table 4**. The results showed that age and foreign language learning history were not correlated with any aspect of foreign language anxiety (*p*s>.05). However, language exposure was negatively correlated with evaluation anxiety (*r*=-.374, *p*=.011) and test anxiety (*r*=-.326, *p*=.027), which indicates that the more foreign language exposure the learners have, the less anxiety of evaluation and test they will experience. Finally, L2 proficiency (indicated by objective verbal fluency test scores) was negatively correlated with communication anxiety (*r*=-.433, *p*=.003) and overall anxiety (*r*=-.357, *p*=.015). These results suggest that the higher L2 (foreign language) proficiency learners have, the less anxious they will be in general, particularly when communicating with others.

**Table 4:** Pearson correlations between linguistic background and FLA.

	1	2	3	4	5	6	7	8	9	10
1. Age	1	.305*	-.111	-.013	-.044	-.046	-.028	.044	.028	.012
2. L2 History		1	.163	.127	.075	-.195	-.216	-.189	-.108	-.271
3. L2 Exposure			1	.315*	-.022	-.011	-.115	-.374*	-.326*	-.253
4. L2 Proficiency (self-rated)				1	.307*	-.084	-.358*	-.024	-.053	-.232
5. L2 Proficiency (verbal fluency)					1	-.226	-.433**	-.191	.154	-.357*
6. General Anxiety						1	.401**	.335**	.223	.717**
7. Communication Anxiety							1	.491**	-.069	.855**
8. Evaluation Anxiety								1	-.056	.706**
9. Test Anxiety									1	.209
10. Overall Anxiety										1

*Correlation is significant at the 0.05 level (2-tailed).

**Correlation is significant at the 0.01 level (2-tailed).

Secondly, we conducted correlation analyses between cognitive control performances and foreign language anxiety. Cognitive control performances included inhibition from the Flanker task and completed categories from the WCST. The results are presented in **Table 5**. As evident from the table, the inhibition [(RT differences between incongruent and congruent trials)/congruent trials] of the Flanker task exhibited a negative correlation with general anxiety, albeit with marginal significance (*r*=-.292, *p*=.054), which suggests that weaker ability of inhibition (higher score) is associated with lower anxiety. Conversely, the mental set shifting (completed categories) of the WCST demonstrated a significantly positive correlation with test anxiety (*r*=.409, *p*=.005), which indicates that better shifting ability is related to higher test anxiety.

**Table 5:** Pearson correlations between Flanker task and the WCST performances and FLA.

	1	2	3	4	5	6	7
1. Inhibition	1	.048	-.292	.175	.181	.089	.081
2. Mental Set Shifting		1	.054	-.093	-.135	.409**	-.011
3. General Anxiety			1	.401**	.335*	.223	.717**
4. Communication Anxiety				1	.491**	-.069	.855**
5. Evaluation Anxiety					1	-.056	.706**
6. Test Anxiety						1	.209
7. Overall Anxiety							1

*Correlation is significant at the 0.05 level (2-tailed).

**Correlation is significant at the 0.01 level (2-tailed).

### Multiple regression analyses

Based on the correlations, we conducted stepwise multiple regressions, to explore what factors can significantly predict Foreign Language Anxiety when all the significant variables of the correlation analyses were entered. Although less theory-driven, stepwise multiple regression is a beneficial statistical method used to identify the best subset of predictor variables to include in a regression model for predicting a dependent variable. It’s an iterative process that involves both adding or removing variables based on their statistical significance (60, 61). For our analysis, L2 proficiency, L2 exposure, inhibition, and mental set shifting are taken as independent variables, and general anxiety, communication anxiety, evaluation anxiety, test anxiety, and overall anxiety are taken as dependent variables. In each regression analysis of each dependent variable, the model begins with no predictor variables. Then, enter the most significant variable; the variable with the strongest correlation is added to the model. Next, the algorithm continues by iterative adding and removing. In the adding process, it considers the remaining predictor variables and adds the one that improves the model’s fit the most while remaining statistically significant. In the removing process, if a variable in the model no longer significantly contributes to the prediction, it is removed. The process continues until all significant variables are included and all non-significant variables are removed (Criteria: Probability-of-*F*-to-enter <= .050, Probability-of-*F*-to-remove >= .100).

The overall results of multiple regression analyses are presented in **Table 6**. The outcomes of the multiple regression analysis conducted on general anxiety revealed that no predictors were retained in the model. This suggests that none of the variables, including L2 proficiency, L2 exposure, and cognitive control, were able to predict general anxiety levels. The results of multiple regressions on communication anxiety showed that both L2 proficiency and inhibition significantly remained in the model, *R*=.516, *R* ^2^=.266, adjusted *R* ^2^=.230, *F* (2, 41) =7.425, *p*=.002, whereas L2 exposure and mental set shifting were excluded (*p*s>.05). According to the model, communication anxiety can be predicted by L2 proficiency and inhibition, with communication anxiety = 42.781– 0.412*L2 proficiency + 24.310*Inhibition + ϵ.

**Table 6:** Results of multiple regressions on different aspects of FLA.

Model ^a^	Unstandardized Coefficients		Standardized Coefficients	*t*	*Sig*.
	B	Std. Error	Beta		
(Constant)	42.781	3.676		11.638	.000
L2 Proficiency	-.412	.117	-.482	-3.530	.001
Inhibition	24.310	10.936	.304	2.223	.032
a. Dependent Variable: Communication Anxiety					
**Model ^b^**					
(Constant)	19.380	1.026		18.883	.000
L2 Exposure	-.073	.029	-.358	-2.488	.017
b. Dependent Variable: Evaluation Anxiety					
**Model ^c^**					
(Constant)	7.426	.666		11.151	.000
Mental Set Shifting	.264	.090	.411	2.923	.006
c. Dependent Variable: Test Anxiety					
**Model ^d^**					
(Constant)	104.925	6.725		15.603	.000
L2 Proficiency	-.538	.210	-.368	-2.562	.014
d. Dependent Variable: Overall Anxiety					

The upper case indicates different regression models conducted. a, b, c, and d correspond with communication anxiety, evaluation anxiety, test anxiety, and overall anxiety respectively as dependent variable.

The results of multiple regressions on evaluation anxiety showed that in the model only L2 exposure predicted the level of evaluation anxiety, *R*=.358, *R* ^2^=.128, adjusted *R* ^2^=.108, *F* (1, 42) =6.192, *p*=.017, whereas L2 proficiency, inhibition and mental set shifting were excluded (*p*s>.05). Therefore, the model indicates that evaluation anxiety = 19.380 – 0.073 * L2 exposure + ϵ.

The results of multiple regressions on test anxiety showed that only the variable of completed categories significantly predicted the level of test anxiety in the model, *R*=.411, *R* ^2^=.169, adjusted *R* ^2^=.149, *F* (1, 42) =8.545, *p*=.006, while L2 proficiency, L2 exposure and inhibition were excluded (*p*s>.05). Therefore, according to model, it indicates that test anxiety = 7.426 + 0.264* completed categories + ϵ.

The results of multiple regressions on overall anxiety scores showed that only L2 proficiency significantly predicted the total score of anxiety in the model, *R*=.368, *R* ^2^=.135, adjusted *R* ^2^=.115, *F* (1, 42) =6.564, *p*=.014, with L2 exposure, inhibition and mental set shifting excluded (*p*s>.05). The model shows that Overall Anxiety = 104.925 – 0.538 * L2 proficiency + ϵ.

In summary, our research findings indicate that various factors distinctly influence different dimensions of foreign language anxiety. More specifically, second language proficiency was a significant predictor of both communication anxiety and overall anxiety. Exposure to the second language significantly predicted evaluation anxiety. Additionally, inhibition was a significant predictor of communication anxiety, and mental set shifting was a significant predictor of test anxiety.

## Discussion

The present study delved into the multifaceted roles of L2 proficiency, L2 exposure, and cognitive control differences in shaping Foreign Language Anxiety (FLA). The findings revealed that these three factors uniquely contribute to distinct aspects of FLA, aligning with our premise that FLA is a complex phenomenon predictable by a diverse array of influences, encompassing linguistic proficiency, language exposure, and cognitive control abilities that transcend linguistic barriers. These findings provide implications in devising targeted interventions that aim to alleviate FLA and enhance language learning outcomes.

Firstly, our findings indicate that higher L2 proficiency mitigates foreign language anxiety, which is consistent with prior research (6, 19, 21, 22, 62). The results of stepwise multiple regressions analyses reveal that L2 proficiency is a pivotal predictor of communication and overall anxiety, with higher L2 proficiency contributing to lower level in the aspects of communication and overall anxiety, suggesting its targeted influence on specific anxiety domains. Notably, our study uncovers that L2 proficiency does not uniformly impact all facets of FLA (e.g., general, evaluation, and test anxiety), particularly distinguishing itself from the communicative and overarching anxiety aspects.

Given that communication anxiety constitutes a significant portion of overall FLA, the precise nature of this relationship remains ambiguous—whether it stems solely from communication anxiety or a confluence of factors. Notably, previous studies, relying primarily on the FLCA to gauge overall anxiety, have overlooked the intricate interplay between L2 proficiency and the discrete components of FLA, namely, general, communication, evaluation, and test anxiety. In addition, a distinguishing characteristic of our study lies in the utilization of the category verbal fluency test as a measure of L2 proficiency, which necessitates not just linguistic proficiency but also language regulation skills (34, 63). This nuanced approach implies that heightened linguistic skills coupled with adept language regulation strategies collectively contribute to a reduction in anxiety levels, particularly in the communication and overall anxiety, providing a fresh perspective on the multifaceted nature of L2 proficiency and its anxiety-mitigating effects.

Secondly, our investigation identifies the pivotal role of language exposure in shaping foreign language anxiety. Our discovery that L2 exposure significantly forecasts evaluation anxiety aligns with prior research, specifying that higher L2 exposure contributes to lower evaluation anxiety. Masangya’s (8) study, for instance, explored the intricate relationship between FLA, L2 exposure, and writing performance among university students in Manila, employing the LEAP-Q, FLCAS, and a writing task. Its findings echoed ours, highlighting the significance of L2 exposure in predicting anxiety, particularly evaluation anxiety.

Literature attests to the beneficial effects of multilingual exposure on communication proficiency (64), suggesting that heightened language exposure is likely to alleviate language anxiety. In our review, we noted that language exposure manifests in diverse forms, ranging from overseas experiences, immersion classes, to early age of acquisition (27, 29). While our participants lacked these immersive experiences, their modest L2 exposure—averaging 32.17% across listening, speaking, reading, and writing activities—still emerged as a notable predictor of FLA, particularly of evaluation anxiety. This finding highlights the importance of even modest L2 exposure in mitigating anxiety, particularly in evaluative context. It implies that as L2 exposure increases, learners are likely to experience reduced anxiety related to others’ evaluations, fostering a more confident approach to language learning situations and lessening the fear of negative assessments.

Thirdly, our findings underscore the intricate interplay between cognitive control mechanisms and anxiety levels, particularly for the components of inhibition and mental set shifting. Specifically, we observed that inhibition significantly predicted communication anxiety, whereas mental set shifting notably predicted test anxiety. This finding aligns with previous research (40–42), highlighting the close nexus between cognitive control and anxiety.

However, the intricacies of this relationship remain somewhat controversial. Firstly, our study revealed that higher inhibition score measured by the Flanker (lower inhibitory control ability) predicted higher level of communication anxiety, which is consistent with previous findings that stronger ability of inhibition reduces anxiety (41, 42). The distinction is that our research uncovered the impact of inhibition on communication anxiety whereas their studies identified the link between inhibition and overall anxiety. However, other investigations, such as Cardinale et al. (65), have reported contrasting results, with individuals exhibiting higher anxiety displaying enhanced inhibitory control capabilities in cognitive tasks, suggesting that elevated anxiety may, paradoxically, coincide with improved inhibitory control, which is actually consistent with our correlation result between inhibition and general anxiety. The distinct outcomes indicating that inhibition is associated with general anxiety and communication anxiety in contrasting manners underscore the significance of discerning the mechanisms underlying various facets of anxiety.

Secondly, in contrast to our hypothesis, increased mental set shifting ability, as indicated by more completed categories of WCST, significantly predicted heightened test anxiety, indicating that better ability of mental set shifting contributes to higher test anxiety. This finding diverges from some previous studies (41, 42), which reported direct links between cognitive control deficits and increased anxiety. Kertz et al. (66), for instance, traced the developmental trajectory of anxiety from early childhood, linking cognitive control deficits to heightened anxiety through school years. Our results, however, indicate that a more proficient mental set shifting ability is associated with heightened anxiety. These inconsistent findings challenge our initial hypotheses and underscore the multifaceted nature of cognitive control and anxiety, particularly in the context of language learning and testing, where learners’ attitudes and strategies may influence their cognitive performance. Rezazadeh and Zarrinabadi (67) propose that, in language learning scenarios, a strong inhibition coupled with a heightened need for closure might hinder learners’ engagement, whereas in testing situations, superior attention control instead of shifting might be crucial for task focus.

Furthermore, recent studies in diagnosed PD patients (68) and neural measures (69, 70) have underscored the elevated risk of cognitive impairment among anxious individuals. However, the directionality of this relationship remains elusive, with debates raging over whether cognitive control influences anxiety or vice versa (71–73). Even so, our findings contribute to the ongoing discourse on the complex interplay between cognitive control and anxiety, emphasizing the need for nuanced examinations of these relationships within specific contexts and considering the diverse dimensions of both constructs. Future research should delve deeper into these nuances, elucidating the precise mechanisms underlying this intricate relationship.

Finally, the findings provide some implications regarding the multifaceted roles of L2 proficiency, L2 exposure, and cognitive control in shaping Foreign Language Anxiety (FLA). By identifying specific aspects of FLA that are influenced by these factors, educators can design targeted interventions tailored to the individual needs of students. Those with lower L2 proficiency may benefit from additional language practice and exposure. The role of L2 exposure in influencing FLA highlights the importance of providing learners with rich and meaningful language immersion experiences. This could involve increased opportunities for interaction with native speakers, immersion programs, or the use of technology to simulate real-life language contexts. By fostering a supportive and immersive learning environment, educators can help reduce anxiety and enhance language acquisition. Recognizing the impact of cognitive control on FLA underscores the need to incorporate cognitive training exercises into language curricula. This could include activities that improve attention, working memory, and inhibitory control—all of which are critical for effective language processing. By strengthening these underlying cognitive abilities, learners may be better equipped to manage anxiety and perform at a higher level in language tasks.

### Limitations and suggestions for future research

The current study presents compelling evidence that foreign language anxiety (FLA) is a multifaceted phenomenon influenced not solely by language proficiency but also by language exposure and cognitive control, each contributing uniquely to distinct aspects of anxiety. However, it is crucial to acknowledge the limitations of this research. Firstly, we are aware of the limitation of stepwise procedures as it is less theory driven than other methods (74). Moreover, the causal link between cognitive control and FLA remains inconclusive and merits further experimental scrutiny in subsequent studies. Longitudinal experiments are essential to unravel the directionality and dynamics of this relationship. Secondly, the current sample size, while informative, could have benefited from an even larger cohort to strengthen the robustness of the multiple regression analyses and enable more nuanced insights. This highlights the need for future investigations to expand their scope and sample diversity to ensure comprehensive understanding. In light of these limitations, future research endeavors must delve deeper into the intricate interplay between cognitive control and FLA. Longitudinal designs should be prioritized to clarify the causal chain and examine how cognitive control evolves and influences FLA over time. Furthermore, a nuanced examination of the various components of cognitive control (e.g., attention, working memory, inhibitory control) and their specific relationships to different facets of FLA is warranted. This should be conducted in tandem with an exploration of additional factors, such as learners’ attitudes and emotions towards language information processing, to provide a more holistic understanding of FLA and its mitigation strategies.

## Conclusion

In conclusion, the present study aimed to unravel the intricate web of factors that contribute to foreign language anxiety, focusing specifically on learners’ language proficiency, language exposure, and cognitive control abilities. Our findings underscore that the multifaceted nature of FLA transcends mere language proficiency; it is intricately linked to the extent of language exposure and individual variations in cognitive control capabilities. These insights are pivotal for comprehending the underlying mechanisms and origins of FLA, offering valuable guidance to language educators and learners alike.

## Data Availability

The datasets presented in this study can be found in online repositories. The names of the repository/repositories and accession number(s) can be found below: https://osf.io/vpzrd/doi.10.17605/OSF.IO/VPZRD.
